# Hypermetabolism and Lipid Alterations Postburn: A Cardiovascular Perspective

**DOI:** 10.1155/cdr/5983391

**Published:** 2026-02-06

**Authors:** Mohammed AbuBaha, Ameer Awashra, Bara AbuBaha, Anwar Zahran, Mohammad Bdair, Dana Sandouka, Sarah Saife, Bara Sawalmeh, Amr Awad, Abdalhakim Shubietah

**Affiliations:** ^1^ Department of Medicine, An-Najah National University, Nablus, State of Palestine, najah.edu; ^2^ Department of Medicine, Advocate Illinois Masonic Medical Center, Chicago, Illinois, USA, advocatehealth.com

**Keywords:** adipose browning, cardiovascular risk, catecholamines, dyslipidemia, endothelial dysfunction, hypermetabolism, lipid remodeling, metabolomics, myocardial remodeling, postburn care

## Abstract

Severe thermal burns involving ≥ 20% of total body surface area (TBSA) initiate a distinct, prolonged physiological cascade extending well beyond the acute phase. This dysregulated response features chronic hypermetabolism, lipid remodeling, and sustained cardiovascular stress. While survival has improved with advances in acute care, the long‐term cardiometabolic effects, particularly the link between lipid abnormalities and cardiovascular risk, remain underexplored. This review highlights the complex pathophysiology of burn‐induced hypermetabolism, including elevated resting energy expenditure, catecholamine‐driven lipolysis, mitochondrial uncoupling, and maladaptive adipose browning. Even in metabolically healthy individuals, these mechanisms promote atherogenic dyslipidemia, characterized by hepatic steatosis, elevated small‐dense LDL, reduced HDL‐C, and persistent hypertriglyceridemia. Emerging lipidomic and clinical data correlate these changes with increased Framingham risk scores, systemic inflammation, and TBSA extent. Simultaneously, cardiovascular vulnerability increases due to myocardial remodeling, autonomic dysfunction, and vascular impairment, particularly in young survivors with prolonged metabolic responses. Imaging and metabolomics reveal endothelial injury, subclinical cardiac dysfunction, and elevated arrhythmogenic risk persisting years after healing. We evaluate current interventions, *β*‐blockers, omega‐3 fatty acids, statins, anti‐inflammatory agents, and structured rehabilitation, within a multimodal framework. Additionally, we identify critical gaps, including the need for precision metabolic modulation, omics‐based monitoring, and tailored cardiovascular risk algorithms. Recognizing severe burns as systemic illnesses with delayed but measurable cardiovascular consequences requires a paradigm shift in long‐term care. This review advocates for proactive, multidisciplinary cardiometabolic surveillance as an essential component of postburn recovery. This review follows the TITAN 2025 guideline for transparency in research and reporting.^1^

## 1. Introduction

Severe burn injuries that cover 20% or more of the total body surface area (TBSA) cause a strong and long‐lasting systemic reaction that goes beyond local tissue destruction and leads to broad hormonal, metabolic, and inflammatory dysregulation. This reaction involves changes in inflammation, metabolism, and hormonal signaling that persist long after injury resolution [1]. A protracted “flow” phase, characterized by increased resting energy expenditure (REE), catecholamine surge, insulin resistance, and persistent catabolism follows the initial “ebb” phase of hypometabolism and shock in the pathophysiological trajectory, lasting months to years after injury. This hypermetabolic response is primarily driven by sustained elevations in catecholamines, cortisol, and glucagon, which collectively amplify lipolysis, gluconeogenesis, and proteolysis [1, 2].

Among the most critically affected systems is lipid metabolism, which becomes profoundly dysregulated under the influence of sustained adrenergic and inflammatory stimuli. Saturated (SFA), monounsaturated (MUFA), and polyunsaturated fatty acids (PUFA) are among the circulating free fatty acids (FFAs) that often increase fourfold acutely in response to adrenergic stimulation, a process that also causes lipolysis and mobilizes FFAs from adipose reserves. These levels correlate with burn severity [3]. Abdullahi et al. found that “browning,” or the change from white adipose tissue to beige fat, that happens after a burn, enhances lipolytic activity and metabolic expenditure. This leads to systemic lipid dysregulation and hepatic steatosis [2, 4].

Lipidomic profiling has indicated that triglycerides and cholesterol levels were usually higher in adults who had burns. Changes in the fatty acid content were associated with adverse outcomes, such as an increased risk of heart disease [3]. A cross‐sectional study found that those who had survived burns had higher levels of triglycerides, low‐density lipoprotein (LDL), and TC/HDL ratios. These factors were also strongly associated with both the TBSA and the Framingham cardiovascular (CV) risk scores [5]. When high‐density lipoprotein cholesterol (HDL‐C) levels remain low and SFAs like stearic and palmitic acids grow, these dyslipidemic patterns become worse. These acids are associated with problems with the endothelium and changes in blood vessels that cause inflammation [2, 6].

Changes in lipids that last for a long time after a burn may be bad for the heart and blood vessels because they can cause atherogenesis, put stress on the heart, and affect metabolism. Burns may make insulin resistance worse, which is partly caused by lipotoxicity. This makes endothelial damage worse and stimulates cardiac remodeling [1, 6]. Even while acute burn treatment has improved, not much is known about how it affects the heart and blood arteries in the long term. This highlights how crucial it is to have targeted treatment for disorders that affect metabolism and lipids.

In this review, we examine how postburn hypermetabolism and lipid derangements affect each other, with emphasis on their mechanistic and clinical CV implications in both terms of how they work and how they affect people in real life. To improve long‐term outcomes for burn survivors, it is important to understand these correlations so that tailored treatments may be made.

## 2. Hypermetabolism After Major Burns

### 2.1. Definition and Phases of Burn‐Induced Hypermetabolism

Severe thermal injury involving roughly one‐third of total body‐surface area or more precipitates a metabolic trajectory that dwarfs the ordinary surgical stress response. Indirect calorimetry studies show REE rising in proportion to burn size; patients with < 10% TBSA burns remain near predicted norms, whereas those exceeding 40% TBSA routinely double baseline requirements and may reach 180% of Harris–Benedict estimates during acute admission [7, 8]. Although absolute values peak within the first month, the curve decays only slowly; even after full epithelial closure, REE stays 20%–30% above predicted for 6–12 months and seldom returns to baseline before the second postburn year [8]. This protracted elevation underpins the distinctive catabolic burden of major burns and provides the physiologic rationale for aggressive early excision, thermoregulation, high‐protein feeding, *β*‐blockade, and other anticatabolic strategies [8].

The initial ebb phase, beginning minutes after injury and lasting approximately 24–72 h, is dominated by shock physiology rather than overt catabolism. Plasma volume plummets as capillary leak peaks, prompting vasoconstriction; bradymetabolism and oxygen consumption may fall 10%–20% below predicted, an adaptive attempt to safeguard perfusion of the heart and brain while resuscitation proceeds [8, 9]. Cardiac output, body temperature, and mitochondrial ATP production are depressed, yet counter‐regulatory hormones surge: catecholamines, cortisol, and glucagon rise within hours, setting the endocrine preconditions for the forthcoming hypermetabolic swing. Recent molecular work links endoplasmic‐reticulum stress and XBP‐1 s activation to suppression of gluconeogenesis during this window, suggesting that hepatic substrate conservation is an intrinsic component of the ebb phenotype [10].

Between the third and fifth postburn day, these compensatory mechanisms are released abruptly, ushering in the flow phase. Vascular permeability normalizes, systemic vascular resistance falls, and cardiac output typically exceeds 150% of nonburned levels by Day 4. Concomitantly, REE accelerates in a curvilinear fashion from ≈130% of predicted on admission to peaks of 140%–180% once full sympathetic activation, brown‐adipose thermogenesis, and cytokine signaling converge [7, 8]. The magnitude and persistence of this response are graded: each additional 10% TBSA amplifies peak REE by ~5%, and inhalational injury, sepsis, or delayed grafting further prolongs the plateau. While adult cohorts generally trend back toward 110%–120% of predicted by 12 months, pediatric series document elevations lasting up to 24 months, reflecting higher adrenergic tone and growth demands [8, 11].

If left untreated, the flow phase evolves into a chronic catabolic state marked by negative nitrogen balance, osteopenia, and immunometabolic dysfunction. Muscle protein loss approaches 1% of lean mass per day in the first fortnight and remains measurable for at least 9 months; persistent catecholamine‐driven lipolysis enlarges the liver and distorts lipid profiles, and dysregulated insulin signaling perpetuates fasting hyperglycemia long after acute wounds have healed. Early excision and grafting, stringent thermal neutrality, high‐carbohydrate/high‐protein enteral feeds, propranolol, oxandrolone, and structured resistance exercise each blunt one or more arms of this pathophysiology, but no single modality fully normalizes metabolism [7, 8].

### 2.2. Molecular and Hormonal Drivers

Peak adrenergic signaling is the lightning rod that ignites the postburn metabolic storm. Within hours of injury, plasma norepinephrine and epinephrine climb four to tenfold, and in adults with ≈60% TBSA burns, they remain supraphysiologic for more than a year. Through *β*‐adrenergic/cAMP‐PKA cascades, these catecholamines accelerate lipolysis, stimulate hepatic glycogenolysis, and upregulate thermogenic genes such as *PPARGC1A* and upregulation of uncoupling protein‐1 (*UCP1*) in adipocytes and myocytes. Parallel surges in cortisol (three to tenfold for at least 3 months) amplify the adrenergic drive by increasing *β*‐receptor density, activating ubiquitin–ubiquitin‐proteasome proteolysis in muscle, and antagonizing insulin at postreceptor nodes, thereby funneling amino acids and glycerol into hepatic gluconeogenesis. The net effect is a sustained, hormonally scripted shift toward substrate mobilization and futile cycling that underpins the elevation in REE [12].

The inflammatory arm of the response is equally potent. Danger‐associated molecular patterns released from the wound activate NF‐*κ*B and inflammasomes, prompting macrophages and neutrophils to secrete tumor necrosis factor‐*α* and interleukin‐6 (IL‐6). Serum concentrations of both cytokines rise several‐fold during the “flow” phase and remain elevated throughout rehabilitation. TNF‐*α* impairs insulin signaling via serine phosphorylation of IRS‐1 and drives muscle catabolism through iNOS‐mediated NF‐*κ*B activation, whereas chronic IL‐6 engages JAK‐STAT3 to induce the atrogenes *MuRF-1* and *Atrogin-1* and to repress IGF‐1–Akt anabolic signaling. These cytokines, therefore, transform the transient hormonal fight‐or‐flight signal into a self‐perpetuating catabolic program marked by insulin resistance and negative nitrogen balance [13].

IL‐6 also crosses mechanistic boundaries by acting on mitochondria. In skeletal muscle and hepatocytes, IL‐6–JAK‐STAT3 signaling promotes DRP1‐dependent mitochondrial fission, collapses membrane potential, and increases reactive‐oxygen‐species leakage; antibody blockade of IL‐6 reverses these changes in burn‐serum–treated myotubes. Such fragmentation reduces ATP‐coupling efficiency and obliges cells to oxidize more substrate for the same bioenergetic yield, further elevating systemic energy expenditure [14].

Concurrently, sympathetic catecholamines and IL‐6 converge on adipose depots to trigger a phenotypic switch from white to beige fat. Human and murine studies show multilocular adipocytes, a several‐fold rise in mitochondrial density, and robust UCP1 induction in subcutaneous fat by 7–14 days postburn; pharmacologic *β*‐blockade or IL‐6 knockout attenuates this browning and halves the increase in REE. Activated brown/beige adipocytes dissipate the proton gradient through UCP1, converting > 300 kcal day^1^ into heat in a 40% TBSA burn and thereby accounting for a sizeable share of the hypermetabolic load [12, 13, 15].

These endocrine, inflammatory, and adipose‐centric pathways form a mutually reinforcing network: catecholamines and cortisol sensitize adipose tissue and muscle to cytokine action; (Table 1) IL‐6 augments adrenergic‐mediated browning signals and fractures mitochondria; mitochondrial uncoupling and ROS amplify NF‐*κ*B activation, which feeds back to sustain cytokine output. Disrupting any single node, *β*‐blockade, anti‐IL‐6 therapy, or PPAR*γ* modulation dampens but rarely abolishes hypermetabolism, underscoring the need for combination strategies that target multiple arms of this integrated molecular cascade [12, 14].

**Table 1:** Hormonal, inflammatory, and mitochondrial alterations during the hypermetabolic response, including their time course, metabolic effects, and underlying mechanisms.

**Parameter**	**Time course**	**Effect**	**Mechanism**
Catecholamines	Within few hours, stays high for months	Increase in both lipolysis and thermogenesis	Activation of *β*‐adrenergic receptors
Cortisol	↑ for 3–6 months	Increase in both proteolysis and insulin resistance	Inhibition of insulin signaling
IL‐6/TNF‐*α*	Peak in flow phase, extended elevation	Increase in inflammation with decrease in insulin sensitivity	JAK‐STAT and NF‐*κ*B pathways
Glucagon	Elevated acutely	Increase level of gluconeogenesis	CAMP‐mediated
Mitochondrial activity	Increased uncoupling	Increase level of energy expenditure	Upregulation of UCP1 (uncoupling protein)

### 2.3. Systemic Effects

REE more than doubles during the first week after injury and rarely normalizes before the second postburn year. Prospective calorimetry in 62 adults demonstrated a 200% REE‐to‐BMR ratio even on Day 28; patients with ≥ 60% TBSA burns sustained the highest absolute rates, and nonsurvivors showed significantly greater surges than survivors, underscoring the close link between energy flux and outcome. Although the curve begins to drift downward once grafting is complete, values remain ≥ 20% above predicted for many months, with each additional 10% TBSA adding ≈5% to the metabolic load. Repeated dressing changes, fever spikes, and operating‐room episodes superimpose transient 10%–15% bumps on this already elevated baseline, so nutritional prescriptions must track not only size and depth but also procedural cadence and sepsis status [16]

Accelerated protein turnover extracts its toll within days. Classical isotope‐tracer work showed net negative leg balance equivalent to ≈1% loss of lean mass per day during the first 2 weeks, and modern ultrasonography confirms that quadriceps thickness and rectus‐femoris cross‐sectional area fall sharply unless counter‐measures are applied. In a 2024 randomized trial of adults with ≥ 40% TBSA burns, early combined resistance‐and‐aerobic training begun on Day 7 halved the decline in quadriceps layer thickness over the initial 6 weeks and produced a threefold greater rebound in muscle force between Weeks 6 and 12 compared with standard care, illustrating both the rapidity of atrophy and its modifiability. Even with such interventions, biopsies months later reveal persistent up‐regulation of ubiquitin–proteasome genes and incomplete recovery of fiber cross section, echoing earlier evidence that catabolism can linger for 9 months or longer in severely burned children and adults [17, 18].

Glucose homeostasis is likewise deranged within hours. Catecholamine‐driven hepatic gluconeogenesis, cytokine‐mediated IRS‐1 serine phosphorylation, and mitochondrial stress converge to produce marked insulin resistance, as documented by clamp studies showing up to 60% reductions in skeletal‐muscle glucose disposal. A 2020 mechanistic review mapped this to sustain elevations of cortisol, IL‐6, and ROS with crosstalk between endoplasmic reticulum (ER) and damaged mitochondria, whereas a prospective cohort of 194 children with > 40% TBSA burns demonstrated abnormal oral–glucose‐tolerance indices for the entire 36‐month follow‐up despite otherwise successful rehabilitation. These data confirm that burn‐related insulin resistance is not a transient by‐product of acute illness but a chronic metabolic sequel that outlasts wound closure and demands long‐term surveillance and, where feasible, pharmacologic modulation [19, 20].

## 3. Lipid Alterations Postburn

### 3.1. Lipoprotein Profile Disturbances

Burns trigger profound physiological changes and a pro‐inflammatory state that affect circulating lipoproteins in a way typically associated with long‐term CV risk. Burn patients have low circulating high‐density lipoproteins (HDLs), and increases in proatherogenic low‐density lipoproteins and marked hypertriglyceridemia [21]. A case–control study showed that small‐dense low‐density lipoprotein (sdLDL) particles were significantly elevated in the burn‐injured patient plasma compared with uninjured controls [6]. Another cohort study of 210 burn survivors demonstrated that LDL, triglycerides, and TC/HDL ratios exceeded the desirable levels [22]. A greater %TBSA was associated with a statistically significant elevation of triglyceride levels [5]. (Table 2).

**Table 2:** Changes in lipid profile parameters over time and their associated metabolic and cardiovascular risks.

**Lipid parameter**	**Direction of change**	**Time frame**	**Associated risks**
Triglycerides (TG)	Increase	Acute to chronic change	Steatosis of the liver and atherogenesis
LDL‐C	Increase	From weeks to years	Plaque development, endothelial cell dysfunction
HDL‐C	Decrease	Persistent changes	Anti‐inflammatory protection is lost
FFAs	Increase	Immediate changes	Lipotoxicity, insulin resistance
VLDL	Increase	Subacute changes	Hypertriglyceridemia and increased liver stress

**Figure 1:**
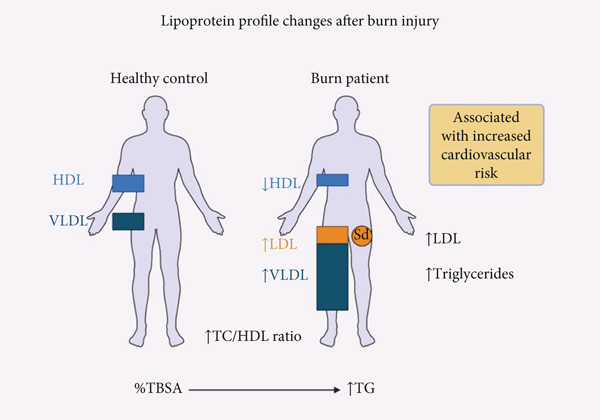
Lipoprotein profile changes after burn injury. This schematic compares the circulating lipoprotein profiles between healthy individuals and burn patients. Burn injury induces significant dyslipidemia characterized by elevated levels of low‐density lipoprotein (LDL), especially small‐dense LDL (sdLDL), very low‐density lipoprotein (VLDL), and triglycerides, alongside a marked reduction in high‐density lipoprotein (HDL). The total cholesterol to HDL ratio (TC/HDL) is also elevated. These alterations are driven by systemic inflammation, cortisol‐induced hepatic VLDL overproduction, and lipoprotein lipase inhibition, all contributing to an atherogenic lipid profile associated with increased cardiovascular risk. Greater total body surface area (TBSA) burned correlates with more pronounced triglyceride elevation.

Cortisol, which is elevated in burned patients, stimulates overproduction of VLDL in the liver, coupled with peripheral inhibition of lipoprotein‐lipase (LPL); the simultaneous HDL fall removes apolipoprotein cofactors needed for VLDL to LDL conversion, leaving remnant particles that further elevate TG [23]. (Figure 1).

### 3.2. Fatty Acid Metabolism

Burns induce lipolysis in white adipose tissue, releasing large quantities of FFAs into the blood. Thermal injury results in mitochondrial dysfunction and endoplasmic‐reticulum stress that blunts *β*‐oxidation capacity. Within hours of injury, *β*‐adrenergic and glucocorticoid signaling plus TNF‐*α*‐dependent MAP‐kinase activation triple the rate of lipolysis in white fat, resulting in a surge of FFAs into plasma [24]. Elevated levels of both palmitic and oleic acids (12.1% increase,*p* < 0.02; 63.0% increase,*p* < 0.0001) in the plasma of burned patients, which suggested increased lipolysis from triglycerides [25].

Serum FFAs and inflammatory markers in burned patients were measured during acute hospital stay in a prospective study. FFAs were acutely elevated postburn and then returned to baseline over time. The study also demonstrated that age and greater burn severity were associated with an impaired acute response in unsaturated FFAs and pro‐inflammatory cytokines. Also, increased levels of SFA and MUFA FFAs are significantly associated with increased mortality [3]. In an obese‐mouse model, thermal injury decreased hepatic *β*‐oxidation proteins and precipitated intracellular lipid with cellular injury, in addition to oxidative stress [26].

### 3.3. Liver Function and Lipid Synthesis

FFA accumulation forced by IL‐6 and hyperinsulinemia leads to activation of SREBP‐1c‐mediated de novo lipogenesis. Human biopsy and imaging studies detect rapid‐onset hepatic steatosis that fits the nonalcoholic fatty liver disease (NAFLD) criteria even in nonobese patients with no prior metabolic disease [26, 27].

The augmented ER stress and inhibition of Akt‐mTOR signaling dysregulated calcium homeostasis, contributed to the decrease of ER–ER‐ER‐ER‐ER‐ER‐ER‐ER‐mitochondria contact, and reduced mitochondrial *β*‐oxidation in high‐fat fed and burned mice, leading to significant hepatic fat infiltration and liver damage, and correlating with elevated aminotransferases, hypoalbuminemia, and oxidative damage [26].

### 3.4. Brown Versus White Adipose Tissue Roles

Burn induces a switch in the phenotype of the subcutaneous fat from white to beige, with associated characteristics such as increased mitochondrial mass and UCP1 and futile thermogenesis [15]. It has also been demonstrated that catecholamines and IL‐6 play a significant role in this process [15].

A study on a mouse model showed that deletion of IL‐6 and the UCP1 (regulators of burn‐induced WAT browning) completely protected mice from hepatic steatosis after thermal injury. Therefore, treatment of postburn mice with propranolol or an IL‐6 receptor blocker decreased burn‐induced WAT browning and the associated hepatic steatosis [2, 15].

## 4. CV Implications of Hypermetabolism and Dyslipidemia

### 4.1. Inflammation and Atherosclerosis

Severe burns cause a long‐lasting hypermetabolic state and drastic changes in lipid metabolism, both of which have serious effects on the heart [28]. Chronic inflammation and dyslipidemia work together to cause and worsen atherosclerosis in people who are recovering from burn injuries [29, 30].

#### 4.1.1. Chronic Inflammation and Vascular Endothelial Dysfunction

After a burn injury, the body goes into a long‐lasting hypermetabolic state where it uses more energy, breaks down more fat, and has big changes in lipid profiles. There is a decrease in HDL and an increase in LDL subfractions, especially small dense LDL particles, which are known to be very bad for your heart [6]. A long‐lasting systemic inflammatory response, shown by higher levels of pro‐inflammatory cytokines like IL‐6 and tumor necrosis factor‐alpha (TNF‐*α*), and higher glycoprotein acetylation markers (GlycA and GlycB), makes this dyslipidemic environment even worse. These inflammatory mediators make the endothelium work less well, lower the amount of nitric oxide that is available, and make blood vessels more permeable. This makes it easier for monocytes to stick to and get into the arterial wall, which is an important early step in atherogenesis [6, 31].

#### 4.1.2. Acceleration of Plaque Formation

The chronic inflammation that happens after a burn hurts the endothelium and speeds up the growth of atherosclerotic plaques. Inflammatory cytokines and oxidized LDL cause macrophages to come to the vascular intima and become active. These macrophages change their metabolism, moving toward glycolysis and taking in more modified lipids. This causes them to become foam cells, which are a sign of early atherosclerotic lesions [32]. After a burn injury, metabolomic studies have found that certain lipids and inflammatory metabolites, like monoacylglyceride (20:4), are higher and linked to a higher risk of heart disease [33]. The continued rise of small, dense LDL subfractions and the fall of anti‐inflammatory HDL subfractions make the lipid profile more likely to cause atherosclerosis, which raises the risk of plaque formation and growth [32, 33].

### 4.2. Myocardial Stress and Structural Changes

A severe burn injury sets off a chain reaction of metabolic and inflammatory responses that have a big impact on the heart muscle. During the acute phase, the heart goes through a lot of physiological stress, which alters its structure and function in ways that may last long after the initial injury.

One of the most important things that happen to the heart after a major burn is that the heart muscle grows bigger. The main cause of this phenomenon is the long‐term hypermetabolic state and high levels of catecholamines, especially epinephrine and norepinephrine, which make the heart work harder and need more oxygen [8, 34]. The myocardium adapts to these needs by going through hypertrophic remodeling, which means that the size of cardiomyocytes grows and the structure of the heart changes. This hypertrophy may start out as a way to help, but it can lead to long‐term problems. Clinical and experimental evidence shows that burn patients often have both systolic and diastolic dysfunction. For example, even years after the injury, they may have a lower ejection fraction and trouble relaxing their ventricles [34, 35].

There are many factors that contribute to burn‐related heart problems. After a burn, patients often go through a distinct phase called “burn shock.” This phase is marked by low blood volume, low heart output, and depression of the heart muscle. This condition is caused by large changes in fluid levels, more permeable blood vessels, and the release of inflammatory mediators like tumor necrosis TNF‐*α* and interleukins, which have direct negative inotropic effects on the myocardium [11, 36]. The presence of myocardial depressant factors in the blood also makes contractility worse. Experimental studies have shown that the myocardium shows cellular edema, mitochondrial dysfunction, and early signs of myocyte degeneration within hours of severe burn injury. All of these changes contribute to the decrease in cardiac performance that has been observed [37, 38].

Once the acute shock phase is over, the CV system enters a hyperdynamic state, which is marked by a higher heart rate and cardiac output. But this compensatory response does not always bring function back to normal. The ongoing metabolic and inflammatory burden, along with ongoing neurohormonal activation, keeps the heart under stress and may cause chronic remodeling of the heart. Long‐term follow‐up studies of children who survived burns have shown that their heart function continues to decline, which suggests that the initial damage to the heart may have long‐lasting effects [37, 39].

### 4.3. Lipid‐Driven CV Risk

#### 4.3.1. Role of Dyslipidemia in Long‐Term CV Disease Risk

Dyslipidemia is a well‐known risk factor for heart disease (CVD), characterized by elevated blood lipid levels. Increased low‐density lipoprotein cholesterol (LDL‐C), decreased HDL‐C, and increased triglycerides are all linked to atherosclerosis and its complications, such as coronary artery disease, stroke, and heart failure [40–42]. The cumulative exposure to elevated LDL‐C, even at moderate levels, significantly increases the risk of coronary heart disease (CHD) over time. Studies have demonstrated a dose‐dependent relationship between the duration of hyperlipidemia in early adulthood and subsequent CHD risk, emphasizing the importance of early and sustained lipid control to minimize lifetime CV risk [41, 43].

**Figure 2:**
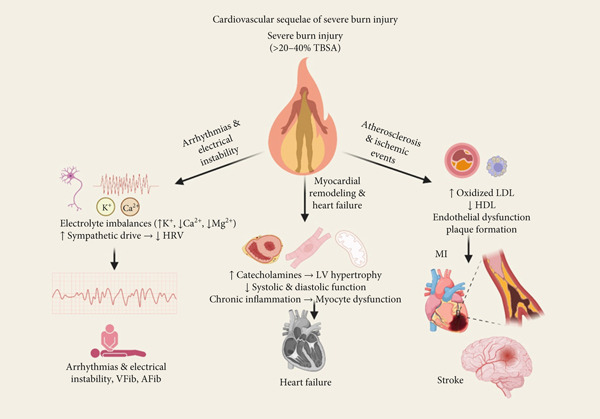
Cardiovascular sequelae of severe burn injury: This figure illustrates the major cardiovascular sequelae that result from severe burn injury. At the center is the initial burn insult, which triggers a cascade of systemic responses—including sustained hypermetabolism, chronic inflammation, and dyslipidemia—that radiate outward into three key pathological pathways [1] atherosclerosis and ischemic events, driven by oxidized LDL, endothelial dysfunction, and plaque formation; [2] myocardial remodeling and heart failure, resulting from catecholamine excess, hypertrophy, and impaired cardiac output; and [3] arrhythmias and electrical instability, due to electrolyte imbalances, sympathetic overactivation, and reduced heart rate variability. Together, these interconnected mechanisms contribute to the elevated long‐term cardiovascular risk observed in burn survivors.

Long‐term follow‐up of individuals with persistent dyslipidemia shows that the risk of CVD is not just based on lipid levels at one point in time but on the total amount of exposure over time. This idea, which is also known as “cholesterol years,” shows that having dyslipidemia early and for a long time puts you at a higher risk of having atherosclerotic events in the future than having it later in life. Because of this, it is recommended that dyslipidemia be aggressively managed to prevent CVD. Statins and other lipid‐lowering therapies have been shown to lower the risk of CV morbidity and mortality [42]. (Figure 2).

#### 4.3.2. Atherogenic Dyslipidemia and Postburn CV Events

Burns cause serious changes in metabolism, such as long‐term hypermetabolism and changes in how lipids are broken down. These changes do not go away quickly; they can last for a long time after the injury and lead to a long‐term state of dyslipidemia and a higher risk of heart disease [6]. After a burn, many patients have a unique lipid profile that includes high levels of small, dense LDL particles, high levels of triglycerides, and low levels of HDL‐C. These are all signs of atherogenic dyslipidemia [5, 6].

Small, dense LDL particles are especially bad for the heart because they can easily get through the arterial wall and are more likely to oxidize, which speeds up the growth of atherosclerotic plaques. Studies of people who survived burns have found that these changes in lipids are linked to a higher risk of ischemic heart disease and other CV events, even when taking into account other known risk factors [5, 6]. Also, the more severe the burn, especially if it covers more than 40% of the TBSA, the higher the risk of major CV events in the years after the injury [22].

Many factors contribute to the pathophysiological mechanisms that increase this risk. Atherogenic dyslipidemia can develop and stay around because of long‐term inflammation, insulin resistance, and activation of the sympathetic nervous system after a burn. Also, the metabolic memory of burn injury, which is shown by long‐lasting changes in the composition and function of lipoproteins, increases the risk of heart disease even more [6].

Because of these results, burn survivors must have regular tests to check their CV risk and lipids. Finding and treating dyslipidemia early in this group may help lower the risk of bad heart outcomes and improve long‐term health [5]. (Figure 2).

### 4.4. Arrhythmias and Electrical Instability

Severe burn injuries raise the risk of heart arrhythmias and electrical instability. This is because of a complicated mix of electrolyte imbalances and autonomic dysregulation. After burns, the body goes into a hypermetabolic state, and the levels of fluids and electrolytes change dramatically. This makes the myocardium more likely to have both short‐term and long‐term electrical problems (Figure 2).

#### 4.4.1. Electrolyte Imbalances and Cardiac Arrhythmias

Burn patients often exhibit significant disturbances in their sodium, potassium, calcium, and magnesium levels, especially during the first few hours after the injury. These imbalances arise from increased capillary permeability, massive fluid shifts, and cellular injury. Hyponatremia is common, especially in the first 24–48 h after an injury, and it often comes with hypoosmolality. Potassium levels may go up at first because cells die and tissue dies, especially in electrical burns. However, they may go down later because of ongoing fluid resuscitation and kidney losses. There are also low levels of calcium and magnesium, which make the risk of arrhythmias even higher [22, 44]. These problems with electrolytes have direct effects on electrophysiology. Hyperkalemia and hypocalcemia are two well‐known causes of life‐threatening arrhythmias like ventricular fibrillation and asystole. Clinical reports show that people with severe hypocalcemia after severe burns have had recurrent ventricular fibrillation. This shows how important it is to keep a close eye on electrolyte levels and fix any problems right away. Patients with large TBSA burns and electrical injuries are at the highest risk because they are more likely to have direct damage to the heart muscle and problems with the conduction system [45–47].

#### 4.4.2. Autonomic Dysregulation and Electrical Instability

Burn injuries cause more than just electrolyte changes; they also cause serious problems with the autonomic nervous system. The hypermetabolic response is marked by a long‐lasting rise in catecholamines, epinephrine, and norepinephrine. These hormones cause persistent tachycardia, an increased need for oxygen in the heart, and increased excitability in the heart [11, 48]. At first, this sympathetic overactivity is helpful, but it can become harmful, making arrhythmias more likely and lowering heart rate variability (HRV).

Studies have shown that having an abnormal HRV in the early postburn period is a strong sign of death, which is caused by the heart′s autonomic system not being able to control its rhythm properly [49]. Burn patients often have lower HRV, which means that their parasympathetic and sympathetic systems are out of balance, and they are more likely to have arrhythmic events. This autonomic imbalance may last for months or even years after the injury, which raises the risk of heart disease in the long term [11, 50, 51].

#### 4.4.3. Clinical Manifestations and Management

Burn patients can have arrhythmias that are benign, like sinus tachycardia and bradycardia, or more serious problems, like atrial fibrillation, ventricular tachycardia, and ventricular fibrillation. Electrical burns are well‐known for causing both immediate and delayed arrhythmias because they damage the heart muscle and the conduction system directly [46, 47]. Continuous cardiac monitoring is recommended for patients with large burns, electrical injuries, or significant electrolyte disturbances.

Management focuses on early recognition and correction of electrolyte imbalances, aggressive resuscitation, and modulation of the hyperadrenergic state. Regular assessment of serum electrolytes, prompt replacement of deficits, and judicious use of beta‐blockers or other agents to temper sympathetic overactivity are essential components of care [52].

## 5. Therapeutic Interventions and Modulation Strategies

Burn‐related cardiometabolic stress is multifactorial, so the best outcomes come from attacking several pathways at once—nutrition, sympathetic overdrive, lipid derangements, systemic inflammation, and deconditioning. Below is a narrative synthesis of the most robust evidence to date.

### 5.1. Nutritional Support Is the Primary Lever

**Figure 3:**
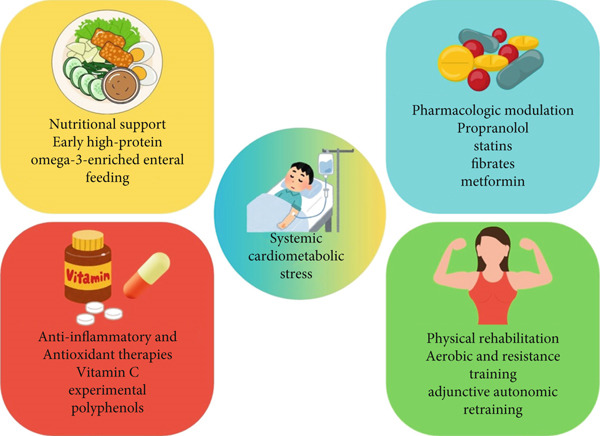
Integrated therapeutic strategies to counter burn‐induced cardiometabolic dysfunction. *This figure illustrates a multimodal approach to mitigating the systemic cardiometabolic stress that follows severe burn injury. Interventions are grouped into four coordinated domains* [1] *nutritional support, emphasizing early high-protein, omega-3–enriched enteral feeding and optimized macronutrient composition;* [2] *pharmacologic modulation, including propranolol, continuation of statins, fibrates for severe hypertriglyceridemia, and metformin when appropriate;* [3] *anti-inflammatory and antioxidant therapies, such as vitamin C and experimental polyphenols (quercetin/resveratrol) with monitoring for oxalate-related risks; and* [4] *physical rehabilitation, progressing from passive movement to aerobic and resistance training with adjunctive autonomic retraining.*

Within the first 6 h after injury, early enteral feeding should begin because it maintains gut integrity and halves the risk of bloodstream infection compared with a delayed start [53]. Caloric prescriptions are typically 25–35 kcal per kilogram per day, roughly 1.5–2 times REE once burns exceed 40% of TBSA. Protein provision must be aggressive: 1.5–2.0 g per kilogram daily in adults and up to 2.5 g in young children, a dose that cuts net nitrogen loss by roughly one‐third and closes donor sites about 3 days sooner [53]. Fat should supply 20%–3% of nonprotein calories, but the qualitative mix matters. Replacing long‐chain SFA fats with medium‐chain triglycerides or structured lipids reduces late hypertriglyceridemia and limits hepatic steatosis [54]. Supplementing 2–3 g of combined EPA and DHA each day attenuates C‐reactive protein by nearly one‐fifth and IL‐6 by about 15% in pooled analyses of randomized trials, although consistent survival or length‐of‐stay benefits have not been demonstrated. A carbohydrate‐to‐lipid ratio near 60:40 keeps serum glucose under 180 mg dL^−1^ while maintaining triglycerides below 250 mg dL^−1^ [55, 56]. (Figure 3).

### 5.2. Pharmacological Modulation Targets the Catecholamine Surge and Lipid Profile

Propranolol is the best‐studied agent: dosing at roughly 0.5–1 mg kg^−1^ every 6 h, titrated to a 15%–20% heart‐rate reduction, lowers REE by almost one‐fifth, trims serum triglycerides by more than 20 mg dL^−1^, and preserves lean mass by 4%–5% in adult randomized trials [57]. In large pediatric cohorts, it also halves the incidence of hypertensive spikes and ventricular arrhythmias without delaying graft take [58]. Statins carry promise because they lower lipids and dampen endothelial activation [59]. Observational data from more than 1800 patients with ≥ 30% TBSA burns indicate that preinjury statin use has been linked to a modest increase in early arrhythmias but a 20% relative reduction in major adverse CV events at 1 year after adjustment for comorbidities [60]. Current practice is therefore to continue any chronic statin once the patient is hemodynamically stable, and to start new therapy only when usual CV indications exist [61]. Fenofibrate—145 mg daily—is reserved for severe hypertriglyceridemia above 500 mg dL^−1^ that persists for a week despite dietary correction. Early pilot work combining low‐dose metformin (500 mg twice daily) with propranolol showed additive reductions in gluconeogenesis and a 22% drop in triglycerides without lactic acidosis, but larger trials are still pending [62]. (Figure 3).

### 5.3. Anti‐Inflammatory and Antioxidant Adjuncts Help Blunt the Chronic Cytokine Drive

High‐dose vitamin C infusions (66 mg kg^1^h^1^ for 24 h) have reduced fluid requirements by about 30% and improved oxygenation indices, though clinicians must monitor for oxalate nephropathy [63]. Curcumin at 2 g orally per day, or as a 2% topical cream, accelerates wound epithelialization and has cut IL‐6 concentrations by more than one‐fifth in small clinical studies; it also offers meaningful pain relief. Resveratrol and quercetin inhibit NF‐*κ*B and oxidative DNA damage in vitro, but human data remain limited, and their use should be restricted to well‐designed trials. Because hepatic microsteatosis can alter drug handling, phased dose escalation and, where possible, therapeutic drug monitoring are prudent [64]. (Figure 3).

### 5.4. Physical Rehabilitation Restores Metabolic Efficiency and CV Resilience

Movement begins with passive and active range‐of‐motion sessions twice daily as soon as the patient is medically stable, typically between Days 3 and 5. Once grafts are secure—often by Day 7 to 10—programs expand to aerobic work at 60%–70% of heart‐rate maximum for 30 min, 5 days a week, combined with progressive‐resistance training [17]. A 12‐week regimen of this nature has raised peak oxygen consumption by about 20%, dropped triglycerides 15%–20%, and increased HDL by roughly 8 mg dL^−1^. Strength work adds another 10%–15% to thigh muscle cross‐sectional area and improves basal metabolic rate efficiency, evidenced by a small decrease in the respiratory quotient. Autonomic retraining, simple heart‐rate–variability biofeedback or yoga breathing for 20 min daily—reduces premature ventricular complexes by nearly one‐third, suggesting partial rebalancing of sympathetic and parasympathetic tone [65]. (Figure 3).

In practice, clinicians should: initiate enteral feedings on the first day; introduce propranolol when tachycardia persists beyond 24 h; add omega‐3 fatty acids or anti‐inflammatories if triglycerides exceed 250 mg dL^−1^ or C‐reactive–protein tops 10 mg L^−1^; start resistance and aerobic training as soon as graft stability permits; and reassess the indication for statins or fibrates upon discharge. By weaving together optimized macronutrients, sympathetic blockade, targeted lipid‐lowering, inflammation dampening, and structured exercise, burn teams can meaningfully blunt dyslipidemia and reduce the long‐term CV burden that too often shadows burn survivors.

## 6. Pediatric Versus Adult Considerations

Pediatric patients exhibit a physiologically elevated metabolic baseline due to ongoing growth. After burn injuries, this metabolic response becomes substantially heightened and prolonged in children, whereas adults usually return to baseline more quickly. Their CV and nutritional systems endure greater physiological demands following burn trauma, which can result in a range of complications if not properly managed [66]. Their systems may eventually become broken down by this continuous strain, leading to nutritional deficiencies, protein breakdown, and muscle loss, all of which may inhibit growth and slow healing. Adults suffer a similar process, but their bodies often recover much more quickly, often in a matter of months [8, 13]. Health issues like diabetes or high blood pressure might make healing more challenging, even though adults may recover more easily. However, they have an advantage in managing the psychological stress of a burn because of their fully developed systems [67, 68].

Burns also affect the body′s ability to process fat. In children, this often results in prolonged disturbances in lipid metabolism, with potential changes in LDL, HDL, and triglycerides. Long after the wounds have healed, these imbalances may persist, raising the chance of cardiac issues and artery obstruction in later life. The burns′ stress and inflammation can also constrict their arteries and increase blood pressure, which can lead to early heart disease. Adults can experience similar lipid‐related complications, though these typically resolve faster unless preexisting health conditions slow recovery. However, especially with a severe burn, even adults may develop heart strain symptoms such as an enlarged heart or irregular rhythms [66].

**Figure 4:**
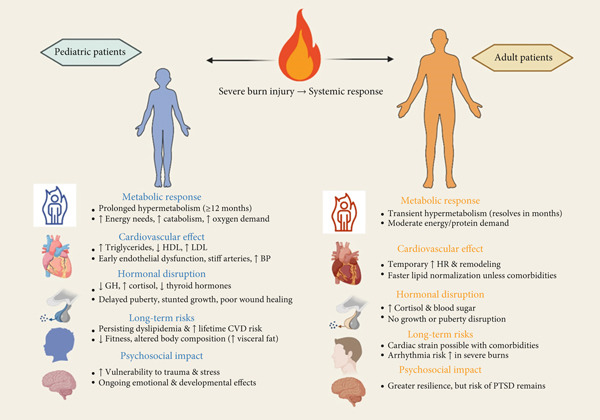
This figure compares the systemic effects of severe burn injury in pediatric versus adult patients. While both groups experience hyper‐metabolism, inflammation, and lipid alterations, children have a longer‐lasting and more disruptive response. Prolonged dysregulation of metabolism, hormones, and cardiovascular function in children increases their lifetime risk of growth impairments and cardiovascular disease. Adults typically recover faster, but outcomes may be worsened by preexisting conditions or severe burn extent.

After a burn, hormones and development are another significant component that distinguishes children from adults, in addition to variations in metabolism and fat processing. Burns to children frequently occur during critical developmental phases. Since their systems are still learning how to balance hormones like stress, thyroid function, and growth hormone (GH), a major injury can upset all of that [66, 67]. This could weaken their bones, delay puberty, and potentially have long‐term impacts on the function of their heart and blood vessels. Adults, however, have already experienced these changes. Hormonal changes, such as elevated stress hormones or blood sugar issues, are still possible, but the long‐term consequences are typically less severe. In addition, the heart may be impacted by the psychological strain that follows a traumatic injury [6, 67]. That type of stress can have a greater effect and make healing more difficult for children, who are still going through a period of mental and emotional development. Burn care must therefore take into account each patient′s age, developmental stage, and emotional needs in addition to their physical needs [66, 68]. (Figure 4).

### 6.1. Differences in Metabolic Response and CV Risk

Because they are still growing and developing, children naturally have faster metabolisms than adults. Following significant burn damage, this baseline rate increases dramatically. While adults typically return to normal metabolic levels within a few months, young kids frequently continue to endure increased metabolic rates for up to a year or even longer [8, 34]. Children′s extended reaction results in increased energy needs and oxygen consumption, insulin resistance and other blood sugar management issues, muscle breakdown and protein loss, and an increased risk of nutrient deficiency [8, 13]. The heart and blood arteries are under more stress as a result of all these alterations. Early indications of CV disease may be brought on by the ongoing stress over time. The CV strain is further increased by persistent inflammation and the generation of dangerous chemicals known as free radicals [6, 67].

The way the body breaks down fats is also affected by burns. Children who have been burned frequently exhibit the following symptoms: a persistent decrease in HDL cholesterol, the “good” cholesterol that aids in blood vessel protection; higher triglyceride levels, which are connected to heart disease and issues with blood vessel function; and a rise in LDL cholesterol, which leads to plaque accumulation in arteries. Young survivors are at an increased risk of developing cardiac issues in the future due to these lipid abnormalities, which can persist even after the burn wounds have healed [34, 66].

In contrast, adult burn survivors typically exhibit a transient metabolic disruption, with a faster return to homeostasis, unless preexisting conditions exacerbate the trajectory. Their fat and cholesterol levels typically return to normal more quickly, but underlying conditions like obesity, diabetes, or high blood pressure can complicate recovery. While adults may experience cardiac strain following burn injury, such as increased heart size or elevated heart rate, their fully developed systems often provide greater capacity to compensate [34, 67]. (Table 3) So, because these alterations can be long‐lasting, children are not only more negatively impacted in the short term but also face a greater risk over longer periods [34].

**Table 3:** Comparison of metabolic and cardiovascular responses between pediatric and adult patients during the hypermetabolic state.

**Parameter**	**Pediatric patients**	**Adult patients**
Hypermetabolic duration	Up to 24 months	Shorter duration about (6–12 months)
Catecholamine levels	Higher levels and peaks	Lower levels
Growth hormone sensitivity	Disrupted and this may be associated with delayed puberty	Stable
Lipid abnormalities	Usually associated with dyslipidemia	Normalize faster
Cardiovascular risk	Higher long‐term risk	Moderate unless others comorbidities are existing

### 6.2. Growth and Developmental Implications

The hormone systems in the body that control growth, metabolism, and heart function are disrupted by burn damage. Since these systems are still developing in children, they are more susceptible to long‐term harm. Important hormones affected include GH, which is usually lower in burn survivors, particularly in children [66, 68]. This can slow down healing and physical development. The hormones glucagon and insulin, which regulate blood sugar, are also affected. After a burn, insulin becomes less effective, leading to elevated blood sugar and improper fat metabolism [13, 66]. Cortisol, often referred to as the “stress hormone,” typically remains elevated following a burn injury. Prolonged high cortisol levels can contribute to fat accumulation around organs, weaken the immune system, and cause muscle breakdown. Thyroid hormones, which help regulate metabolism, may also be disrupted. This slows down the body′s ability to metabolize fats and energy efficiently. Children who experience disruptions in these hormone systems may face limited growth, weakened bones, and delayed puberty. These developmental challenges can also indirectly increase the risk of future heart disease [34, 66].

When the wounds from a severe burn heal, the effects often continue. Recovery can involve several long‐term challenges for many survivors, especially children [34]. Poor CV fitness is common; many pediatric patients still experience diminished heart function and reduced stamina even years after recovery. Stiff arteries and high blood pressure are also early warning signs that can eventually lead to serious heart problems. There are also modifications in body composition [67]. As unhealthy fat accumulates around the organs and muscle mass decreases, this contributes to increased insulin resistance and unfavorable cholesterol profiles [34].

Although adults usually do not face the same growth‐related challenges, they may still experience issues such as abnormal heart rhythms or structural changes to the heart. Older individuals with preexisting health conditions may find recovery more difficult and may be at an even higher risk for developing cardiac complications [13].

## 7. Clinical Monitoring and Risk Stratification

Aside from wound closure, the postburn course exposes survivors to extreme lipid dysregulation and a hypermetabolic state of chronic nature, both of which extend CV risk. Even when total LDL‐C is not elevated, the plasma metabolomic profile shows constant elevation in small dense LDL particles (LDL5/LDL6), which are strongly associated with atherogenesis and CV disease risk [6].

Through several years after a burn, endothelial damage and systemic metabolic stress are sustained by chronic inflammation in the type of cytokines TNF‐*α*, IL‐6, and IFN‐*γ* [1]. Maladaptive stress response is driven by catecholamines and inflammatory mediators that worsen CV load and FFA flow through ongoing lipolysis and browning of adipose tissue [69]. Specifically, even moderately burned survivors have also been discovered to incur higher rates of CV hospitalizations, showing that systemic effects, instead of the size of injury alone, regulate long‐term risk [6]. This finding shows the need for systems to integrate risk assessment, biomarker surveillance, and vascular imaging.

### 7.1. Biomarker Surveillance

To ensure accurate CV diagnosis and effective management in patients who have sustained burn injuries, the use of reliable serum and plasma biomarkers is essential. Lipid profile abnormalities are particularly informative; a 2024 study reported that among 210 adults with burns covering more than 20% of TBSA, 13 patients exhibited a 20% higher risk of developing CV disease based on the 10‐year Framingham risk score. Notably, mean LDL‐C levels were 145 ± 24 mg/dL, and triglycerides reached 225 ± 45 mg/dL, both significantly elevated compared with matched unburned controls and strongly correlated with increased CV risk [22]. High‐sensitivity C‐reactive protein (hs‐CRP) levels, which remained between 3 and 5 mg/L following wound healing, compared with < 1 mg/L in healthy individuals, indicate persistent low‐grade inflammation, a known contributor to endothelial dysfunction and atherosclerotic plaque progression [4]. While cardiac troponins (cTnI/T) may show transient elevation postburn (*r* = 0.18, *p* = 0.12), they do not consistently predict long‐term reductions in left ventricular (LV) ejection fraction. Therefore, troponin levels should be interpreted within the broader context of clinical symptoms and cardiac imaging findings [6]. Collectively, these biomarkers provide crucial insight into burn‐induced CV stress and support early risk stratification and intervention.

### 7.2. Imaging and Functional Testing

Early detection of subclinical CV changes is promoted by an exhaustive imaging strategy. Echocardiography findings in well‐healed burn survivors (BSA 37% ± 12%) from a 2019 PLoS MRI/Echo study showed a 12% ±6% reduction in global longitudinal strain, an efficient sign of subclinical dysfunction, and a mean LV mass at the 5th percentile [6]. A 0.1 mm increase in carotid intima media thickness (CIMT) is associated with a 15%–17% increased risk of myocardial infarction (MI) and stroke; although CIMT is underutilized postburn, it remains an effective predictor that should be employed [70]. Pulse wave velocity (PWV) is another key marker, as a 1 m/s rise is independently linked to a 14% increase in CV event progression; its correlation with hs‐CRP in inflammatory conditions supports its role in burn follow‐up [71]. Additionally, flow‐mediated dilatation (FMD) levels below 8% are indicative of future CV events and serve as an early marker of endothelial dysfunction.

### 7.3. Multidisciplinary Management Approach

Care coordination by specialized teams is essential for providing ideal care. Burn and critical care teams should be responsible for screening, initial biomarker assessment, and transitioning patients to outpatient care. Cardiologists must conduct routine vascular imaging and echocardiography, incorporating these findings into personalized CV risk profiles. Meanwhile, nutritionists and rehabilitation therapists play a key role by implementing anti‐inflammatory dietary plans, such as those rich in omega‐3 fatty acids, and developing structured exercise regimens to restore aerobic capacity, reduce lipid abnormalities, and mitigate inflammation [72].

### 7.4. Suggested Monitoring Timeline (Table 4)

**Table 4:** Proposed timeline for cardiovascular and metabolic assessment and follow‐up after discharge.

**Time point**	**Assessments**
Discharge	Lipid panel (TG, LDL/HDL), hs‐CRP, cTn, BP, HR; baseline echocardiogram
6 Weeks	Repeat biomarkers + echocardiogram
6 Months	Add CIMT/PWV/FMD and update the cardiovascular risk score
12 Months and annually	Ongoing biomarker and imaging review; frequency tailored to risk status

#### 7.4.1. Tiered Risk Management Strategy

Patients are stratified based on CV risk levels: those at low risk (< 10% with normal imaging) require yearly follow‐up and lifestyle modification; intermediate‐risk individuals (10%–20% with early imaging changes) should begin antihypertensive and statin therapy alongside biannual imaging; and high‐risk patients (> 20% with biomarker or imaging abnormalities) need initiation of ACE inhibitors, high‐dose statins, and strict control of blood pressure and glucose, with follow‐up every 3–6 months. Emphasizing chronic inflammation and lipidomic profiling may support the development of burn‐specific CV risk scores, a promising direction for future research.

## 8. Future Directions and Research Gaps

### 8.1. Long‐Term CV Surveillance of Burn Survivors Remains Strikingly Underdeveloped

Most evidence comes from single‐center or short‐horizon studies, yet a 2023 Australian cohort of 1126 adults showed that 13% had an estimated 10‐year atherosclerotic–cardiovascular‐disease (ASCVD) risk > 20% and that dyslipidemia (elevated LDL, triglycerides, and TC/HDL) correlated independently with burn size and years since injury [22]; parallel pediatric work reveals that children retain heightened carotid‐intima thickness and proatherogenic lipid profiles for at least 5 years after wounds have healed [6]. These signals mirror excess late mortality documented in a 2024 matched‐cohort analysis of 18,937 survivors, which reported a hazard ratio of 1.26 for CV death beyond 10 years postburn [73]. To move from signal detection to risk prediction, national and international registries such as the American Burn Association′s Burn Care Quality Platform (BCQP) must evolve to include granular cardiometabolic variables and long‐term follow‐up modules [74].

### 8.2. Therapeutic Pipelines Should Widen Beyond *β*‐Blockers and Statins Toward Agents that Directly Modulate Lipolysis and Insulin Resistance

Negative‐feedback regulators of adipocyte lipolysis, most notably the long–chain‐fatty‐acid receptor FFAR4 (GPR120), are emerging as druggable targets; preclinical activation of GPR120 suppresses catecholamine‐driven free–fatty‐acid release and improves insulin sensitivity without hypoglycemia [75]. Agonists now in Phase‐II evaluation for diabetes and NAFLD could be repurposed for burns once safety is confirmed in hyperadrenergic states [76]. Likewise, fibroblast–growth‐factor‐21 analogues and GLP‐1 receptor agonists offer dual lipid–glucose benefits and warrant mechanistic trials in burn catabolism. Early human data combining metformin with propranolol reduced triglycerides by 22% without lactic acidosis, suggesting that multidrug metabolic checkpoints are feasible [77]. High‐throughput screening has also identified burn‐activated beige‐fat progenitor pathways driven by linoleic acid, pointing to another axis for pharmacologic browning or uncoupling therapies [78].

### 8.3. Precision‐Medicine Approaches Will Hinge on Uncovering Genetic Modifiers of the Inflammatory and Lipid Response to Thermal Injury

Functional polymorphisms in the TNF‐*α* promoter (–308 G/A) and TLR4 variants already influence sepsis risk and long‐term lipid profiles after burns [79]. Genome‐wide association and polygenic‐risk‐score studies, however, have yet to be conducted in large burn cohorts. Integrating host genomics with microbial and environmental exposures could stratify patients into “rapid metabolizers” who need aggressive *β*‐blockade versus “low responders” who may benefit more from anabolic or anti‐inflammatory agents [80].

### 8.4. Systems Biology and Metabolomics Promise to Map the Burn‐Specific Cardiometabolic Signature

Integrated transcriptomic–metabolomic profiling of murine and porcine burn models has already revealed coordinated upregulation of hepatic gluconeogenesis, sphingolipid turnover, and branched‐chain‐amino‐acid catabolism– pathways tightly linked to CV risk [81, 82]. Human serum‐omics studies remain sparse, limited by small sample sizes and heterogeneous time‐points. Large‐scale, longitudinal biobanking coupled with untargeted metabolomics could uncover early biomarkers that predict which transient hypertriglyceridemia will evolve into persistent atherogenic dyslipidemia. Cloud‐based cardiometabolic registries that capture both clinical and omic data, such as the recently launched Veradigm disease‐specific platforms, offer a ready framework for such integrative analyses [83].

Collectively, the field must transition from descriptive studies toward prospective, mechanistic, and gnomically informed trials that translate molecular insights into tailored therapies capable of neutralizing the burn survivor′s hidden CV burden.

## 9. Conclusion

Severe burn injuries cause a multisystemic, intricate reaction that goes well beyond tissue healing and has long‐term effects on CV, inflammatory, and metabolic balance. A chronic hypermetabolic state that causes significant changes in lipid metabolism, including persistent dyslipidemia, adipose tissue browning, hepatic steatosis, and systemic lipotoxicity, lies at the heart of this protracted trajectory. These disturbances raise the long‐term CV risk among burn survivors by aggravating endothelial dysfunction, encouraging the development of atherosclerotic plaque, and causing maladaptive cardiac remodeling.

The chronic cardiometabolic burden of burns is still poorly understood and treated, despite tremendous advancements in acute burn care. Although they provide some advantages, the current treatment approaches, nutritional optimization, sympathetic blocking, anti‐inflammatory techniques, and organized rehabilitation cannot completely reverse the chain reaction of lipid‐driven CV sequelae. The necessity for integrated, longitudinal care models that include risk assessment tools, sophisticated imaging, and biomarker surveillance specific to this group is being underscored by emerging research.

A paradigm change that reframes significant burns as a chronic metabolic condition with systemic ramifications, rather than only a reconstructive issue, is necessary to lessen the hidden CV load that shadows burn survival. Mechanistic investigations, omics‐guided therapies, and precision‐medicine approaches that can restore metabolic balance and maintain long‐term CV health must be given top priority in future research. We can only guarantee that enhanced survival results in long‐lasting health and a higher standard of living for burn survivors by means of such multidisciplinary initiatives.

## Ethics Statement

Ethical approval is not required for this review article.

## Consent

The authors have nothing to report.

## Disclosure

This article was not commissioned and was externally peer reviewed.

## Conflicts of Interest

The authors declare no conflicts of interest.

## Author Contributions

Mohammed AbuBaha and Ameer Awashra contributed equally as co–first authors. Mohammed AbuBaha: conception and design of the review, performed the literature search, synthesized the data, and drafted the initial manuscript. Ameer Awashra: study design, literature review, data interpretation, and manuscript drafting. Bara AbuBaha: data acquisition, literature appraisal, and manuscript writing and editing. Anwar Zahran: data collection, critical review of the literature, and manuscript editing. Mohammad Bdair: literature screening, data interpretation, and drafting of relevant sections. Dana Sandouka: assisted in manuscript preparation, figure conceptualization, and revision of the clinical sections. Sarah Saife: manuscript drafting, critical revision for intellectual content, and corresponding author responsibilities. Bara Sawalmeh: assisted in literature review, data organization, and manuscript revision. Amr Awad: contributed to the interpretation of the manuscript and provided critical revisions of the manuscript. Abdalhakim Shubietah: provided senior supervision, contributed to the conceptual framework, critically reviewed the manuscript for scientific accuracy, and approved the final version.

## Funding

No funding was received for this manuscript.

## Data Availability

This study did not involve the creation or analysis of any new data. Therefore, data sharing is not applicable.
